# A simple three-dimensional stent for proper placement of mini-implant

**DOI:** 10.1186/2196-1042-14-45

**Published:** 2013-11-12

**Authors:** A Sumathi Felicita

**Affiliations:** 1 Department of Orthodontics, Saveetha Dental College, Chennai, Tamil Nadu, India

**Keywords:** Stent, Mini-implant, Three-dimensional control

## Abstract

**Background:**

This paper deals with the fabrication of a three-dimensional stent which is simple in design but provides an accurate placement of a mini-implant in three planes of space, namely, sagittal (root proximity), vertical (attached gingiva/alveolar mucosa) and transverse (angulation).

**Findings:**

The stent is made of 0.018 × 0.025 in. stainless steel archwire which consists of a ‘u loop’ angulated at 20°, a vertical limb, a horizontal limb and a stop. The angulation of the ‘u’ helps in the placement of the mini-implant at 70° to the long axis of the tooth. The vertical height is determined such that the mini-implant is placed at the mucogingival junction. The mini-implant is placed with the aid of the stent, and its angulation and proximity to the adjacent roots are checked with a cone beam computed tomography image. The cone beam computed tomography image showed the mini-implant at an angle of 70° to the long axis of the tooth. There is no contact between mini-implant and the roots of the adjacent teeth.

**Conclusion:**

This stent is simple, easy to fabricate, cost-effective, and provides ease of insertion/removal, and three-dimensional orientation of the mini-implant.

## Findings

### Introduction

The accurate placement of mini-implant is of paramount importance for its stability. Stability depends on a number of factors [1-8]. Proximity to the root surface, placement in the alveolar mucosa, and improper angulation have been attributed to mini-implant failure. Root proximity is a major cause of mini-implant failure [3,5]. Placement in the alveolar mucosa can result in peri-implantitis with failure of the mini-implant.

Root proximity can be reduced by angulating the mini-implant to the long axis of the tooth. This facilitates placement of the tip of the mini-implant towards the root apex. This reduces root proximity as well as increases the contact between the mini-implant and the cortical bone with increased stability of the mini-implant. Hence, a stent was designed to aid in optimum mini-implant placement. A stent is a surgical guide which aids in the proper placement of the mini-implant in the three dimensions of space, namely, sagittal (root proximity), vertical (attached gingiva/alveolar mucosa), and transverse (angulation).

The ideal requirements of a stent are the following:

• Enables placement of the mini-implant at the correct occlusogingival height preferably in the attached gingiva

• Enables accurate mesiodistal placement of the mini-implant away from the roots of adjacent teeth

• Enables an appropriate angulation of the mini-implant to the long axis of the tooth in the transverse plane

• Easy to fabricate and cost-effective

• Ease of placement and removal

• Versatility of use with ease of placement in different areas of the maxilla and mandible

**Figure 1:**
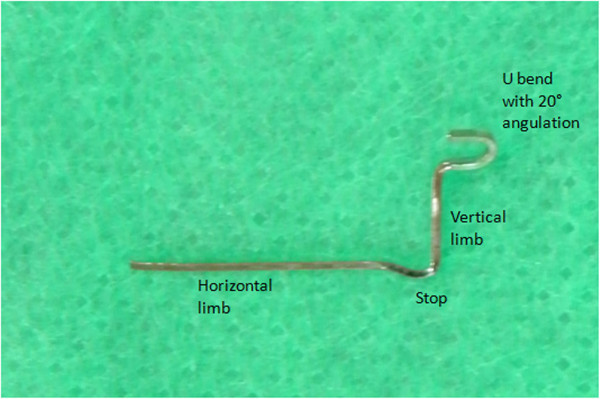
Parts of the stent.

Taking the above factors into consideration, the stent has been fabricated from 0.018 × 0.025 in. stainless steel wire in the present case for placement in the auxillary buccal tube of the maxillary first molar. The stent consists of a ‘u’ loop, a vertical limb, a horizontal limb and a stop (Figure 1).

### Materials and methods

#### Steps in the fabrication of stent

**Figure 2:**
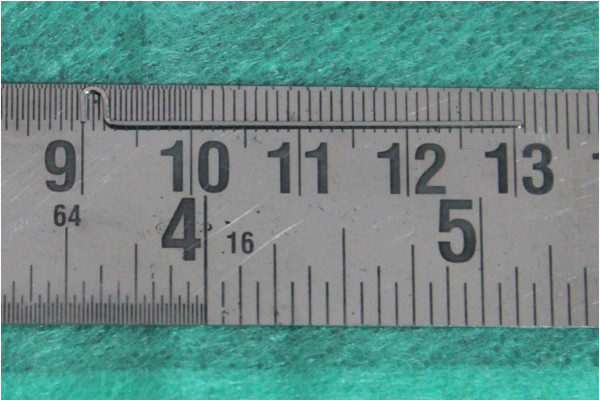
**The u-shaped bend.** U-shaped bend with 2-mm separation between the limb and 90° vertical bend given to the ‘u’ bend.

Stainless steel 0.018 × 0.025 in. is used to fabricate the stent. A ‘u’-shaped bend is placed such that the limbs are 2 mm apart. A 90° vertical bend is placed from the ‘u’ bend (Figure 2).

**Figure 3:**
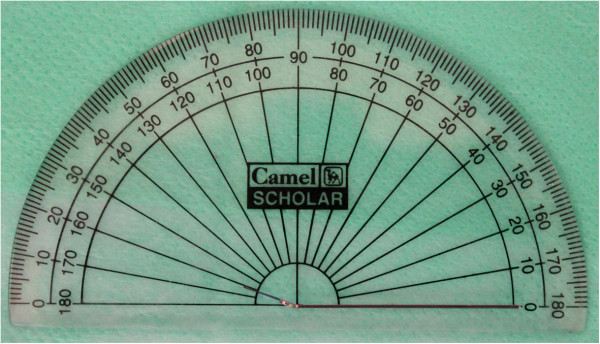
Angulation of ‘u’ at 20°.

The ‘u’ bend is angulated such that it is at an angle of 20° to the vertical leg (Figure 3). The height of insertion and the angulation of the mini-implant depend on the area of mini-implant placement, the anatomic structures at the site of insertion, and biomechanical considerations. In the posterior region, a vertical height of 8 mm from the alveolar crest has been suggested to prevent insertion into the maxillary sinus [9].

Pretreatment orthopantomogram can be used to assess the position of the maxillary sinus, the mental foramen, the mandibular canal and the interradicular bone in the posterior region. In the anterior region, intraoral periapical radiograph can be used to check the width of the interradicular bone.

The treatment mechanics in the present case required application of an intrusive component of force during retraction of maxillary anterior teeth. Hence, the mucogingival junction was chosen for mini-implant placement as it forms the superior limit of the attached gingiva.

**Figure 4:**
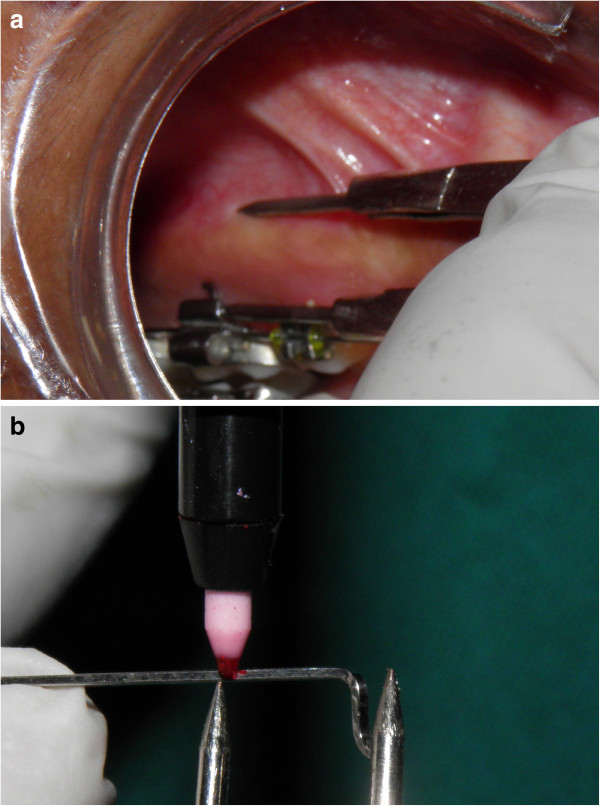
**Measurement of vertical height and marking of the stent. (a)** Desired vertical height was measured and **(b)** was transferred to the stent.

**Figure 5:**
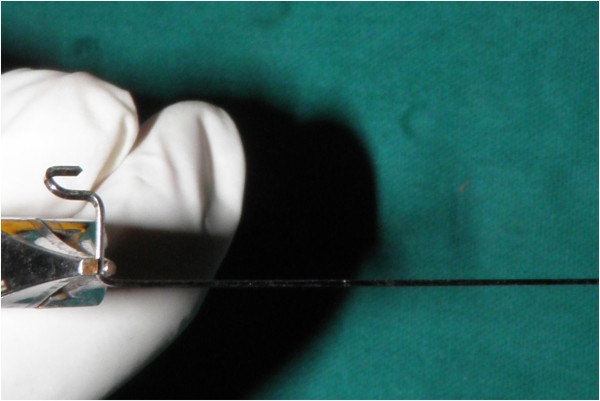
A ‘L’ bend is given.

The vertical height is measured from the mucogingival junction to the auxillary tube of the maxillary first molar tube (Figure 4a).This distance is marked from the superior surface of the ‘u’ loop to the vertical section of the wire (Figure 4b). A ‘L’ bend is given (Figure 5).

**Figure 6:**
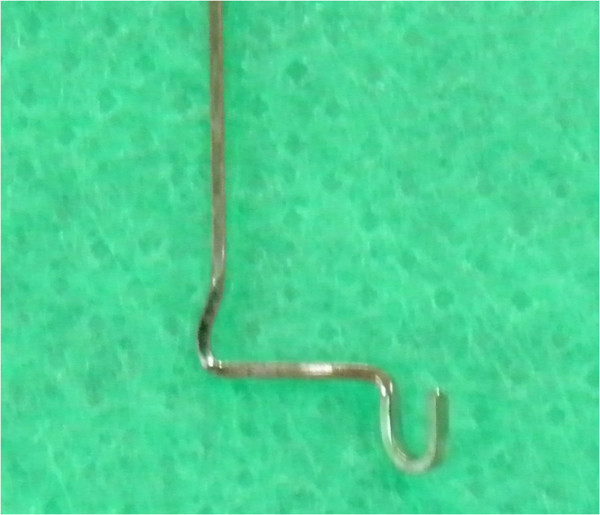
**Placement of the stop.** The stop was placed just anterior to the auxillary tube to control the mesiodistal position of the stent.

**Figure 7:**
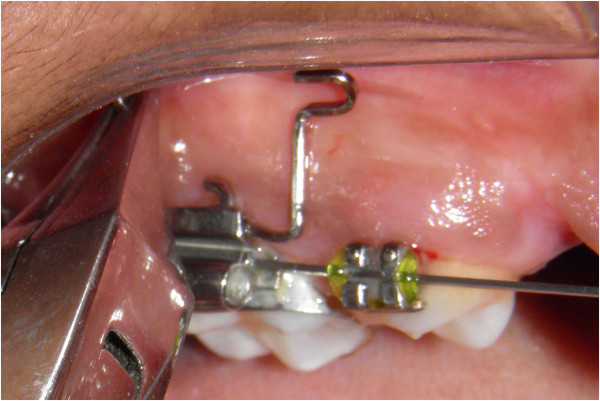
**Placement of the horizontal limb of the stent in molar auxillary tube and cinching.** The horizontal limb passes through the auxillary tube of the molar tube and the stop prevents slippage of the wire into the auxillary tube. The stent was placed in its final position and cinched.

To prevent slippage of the wire and to place the wire at the desired mesiodistal position, a stop is placed just anterior to the auxillary tube (Figure 6). The horizontal leg of the stent is placed in the auxillary buccal tube of the maxillary first molar and cinched distal to it (Figure 7).

It should be noted that the ‘u’ loop is placed such that the opening of the ‘u’ is in the posterior region and the closed section is in the anterior region towards the eye of the operator. This acts a guide during mini-implant placement. If the closed section of the ‘u’ is in the posterior region, the guidance offered by the wire is obscured by the mini-implant and mini-implant driver.

**Figure 8:**
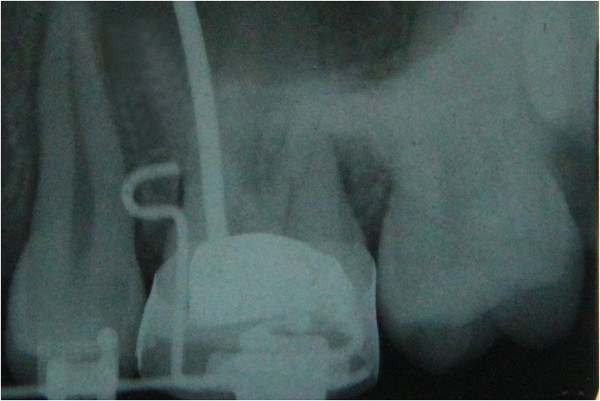
Intraoral periapical radiograph showing the position of the stent in the interradicular bone.

An intraoral periapical radiograph is taken to check the vertical and mesiodistal position of the stent and the exact site of mini-implant placement as determined by the position of the ‘u’ (Figure 8).

Lox* 2% jelly (lignocaine hydrochloride 2%, methyl paraben 0.061%, propyl paraben 0.027%; Neon Laboratories Ltd, Mumbai, India) is used as topical anaesthetic agent. 1 ml of Lox* solution [lignocaine hydrochloride 2% with adrenaline bitartrate (1:200,000)] is injected in the mucogingival sulcus adjacent to the area of mini-implant placement.

**Figure 9:**
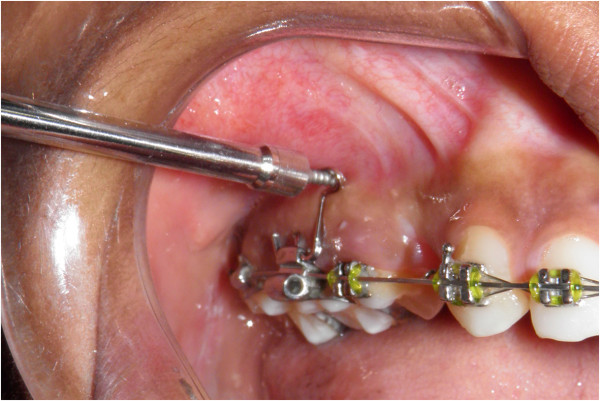
**Initial insertion of the mini-implant perpendicular to the alveolar bone.** Note that the mini-implant is closer to the upper limb of the ‘u’.

**Figure 10:**
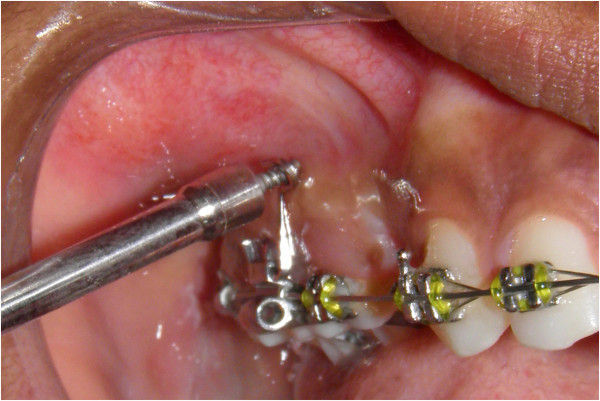
**Change in angulation at 70° to the long axis to facilitate better cortical adaptation.** Note that the mini-implant is parallel to the upper and lower limbs of the ‘u’.

Orlus self-drilling mini-implant (Ortholution, Kyunggi-do, Korea) which is 1.4 mm in diameter and 7 mm in length is first placed perpendicular to the buccal surface for initial penetration. If insertion is attempted at an angle without initial purchase, there is a possibility of mini-implant slipping during insertion. Note the mini-implant abutting the superior surface of the ‘u’ bend (Figure 9). After the initial purchase is achieved, the mini-implant is withdrawn and inserted parallel to the superior and inferior section of the ‘u’ (Figure 10). This will help the clinician to obtain a mini-implant angulation of 20° to the occlusal plane.

The mini-implant is tightened. During the end of placement, interference will be encountered due to the angulation of the ‘u’.

**Figure 11:**
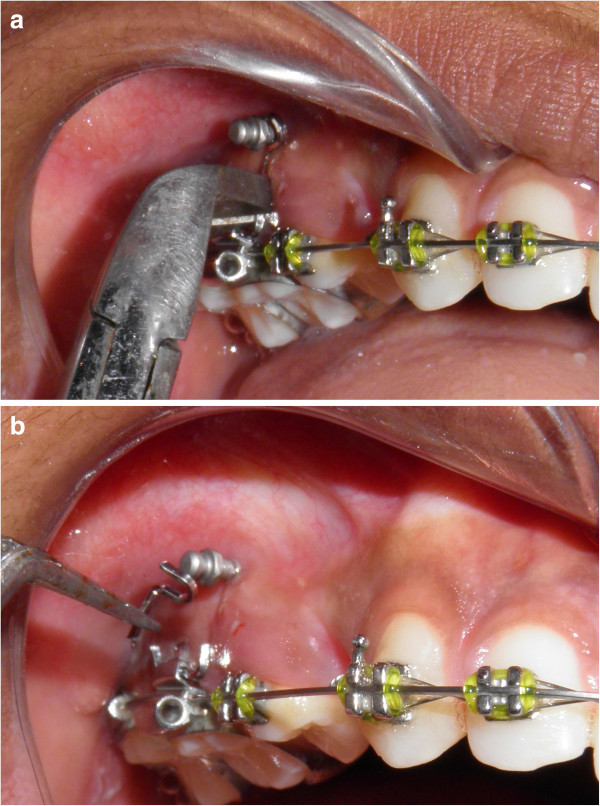
Vertical limb is cut (a) to facilitate complete insertion of mini-implant (b).

The vertical leg is cut and the segment of wire is removed (Figure 11a,b).

**Figure 12:**
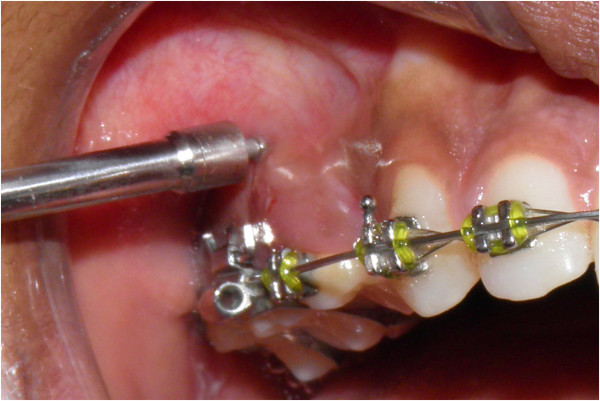
Final tightening of the mini-implant.

**Figure 13:**
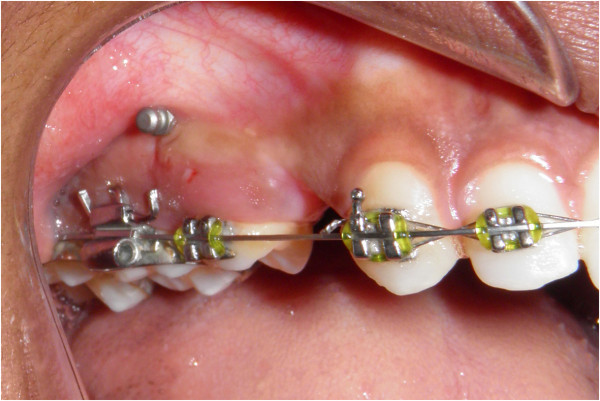
Mini-implant immediately after placement.

Final tightening of the mini-implant is done (Figure 12). Figure 13 shows the mini-implant immediately after placement.

### Results

**Figure 14:**
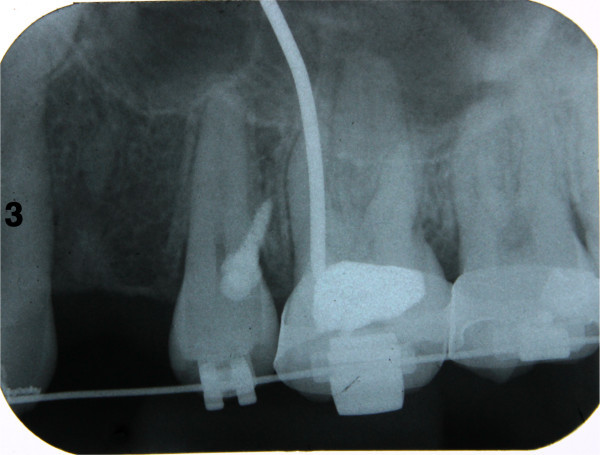
Intraoral periapical radiograph taken to check position of the mini-implant.

Intraoral periapical radiograph is taken to check the position of the mini-implant (Figure 14).

**Figure 15:**
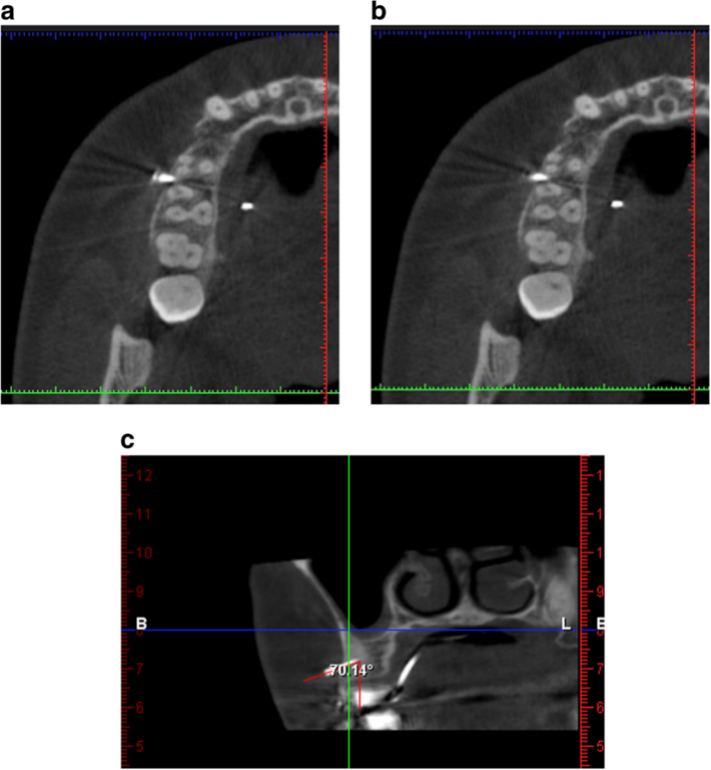
**Images from cone beam computed tomography. (a to c)** Cone beam computed tomography taken to check mesiodistal position and accuracy of angulation. An angulation of 70° to the vertical has been measured.

A cone beam computed tomography is also taken to check the position of the mini-implant in the three planes of space as it is superior to the periapical dental radiograph for assessing root proximity [6]. In the mesiodistal direction the mini-implant was found to be away from the roots of the adjacent teeth (Figure 15a,b). In the transverse plane the angulation of the mini-implant was measured and was found to be 20° (Figure 15c).

#### Modifications of the stent

The stent can be placed at any site using wire of different cross sections depending on the need of the case.

In the maxillary and mandibular anterior region, the stent made of 0.019 × 0.025 in. stainless steel wire can be placed in the bracket slot after removal of the base arch wire. The vertical limb can be contoured to adapt and follow the contour of the labial surface of the maxilla and mandible.

The same can be done if the mini-implant is to be placed in between the maxillary/mandibular premolars or canine region.

**Figure 16:**
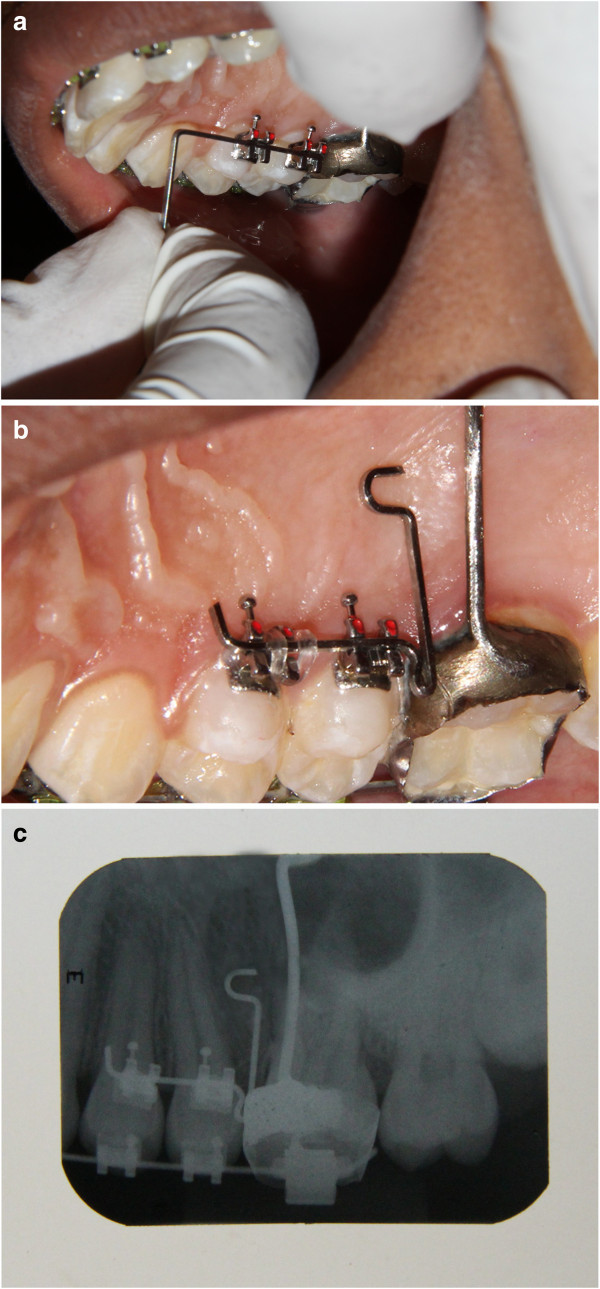
**Placement of 0.019 × 0.025 in. stainless steel wire and stent and imaging after placement. (a)** Wire placed to check alignment of 0.022 in. brackets prior to curing. **(b)** Stent placed in the palatal aspect of the teeth. **(c)** Intraoral periapical radiograph taken after placement of the stent.

The stent can also be used in the palatal aspect in the posterior region. A 0.022-in. bracket is bonded on the palatal aspect of the premolars or a molar buccal tube can be bonded on the palatal aspect of the molar depending on the site of mini-implant placement. Care should be taken to place the brackets in the same plane on the premolars for ease of placement of 0.019 × 0.025 in. stainless steel wire (Figure 16a). A stent made of 0.019 × 0.025 in. stainless steel wire is contoured and ligated in the brackets placed on the palatal aspect (Figure 16b). Figure 16c shows an intraoral periapical radiograph taken after placement of the stent.

In situations where the auxillary tube is not available as in the case of a bondable buccal tube, it is possible to place a 0.019 × 0.025 in. stainless steel wire in the main buccal tube after removal of the base arch wire.

If a 0.018-in. bracket prescription is used, 0.016 × 0.022 in. stainless steel can be used to fabricate the stent.

The contact between the adjacent teeth can be used as a guide for mesiodistal positioning of the mini-implant [10]. During reinsertion of the mini-implant after initial purchase, the mini-implant can be oriented such that it is placed slightly closer to the anterior closed portion of the ‘u’ loop. This will bring the mini-implant more parallel to the contact area of the adjacent teeth.

### Discussion

Several factors have been attributed to the success of mini-implant, namely, mini-implant factors (type, diameter, site of mini-implant placement and length), local host factors (occlusogingival positioning), and management factors (angle of placement, onset and method of force application, ligature wire extension, exposure of mini-implant head and oral hygiene) [2].

The stability of the mini-implant is affected by extreme root proximity rather than the width of the alveolar septum. However, stability of the mini-implant is not greatly affected if there is no periodontal ligament invasion [7].

Buccal and palatal interradicular cortical bone thickness and alveolar process width tend to increase from crest to base of the alveolar process. Hence, the mini-implant should be placed apically to avoid root proximity. The distance between the roots is widest between second premolar/first molar and first premolar/second premolar and the least between central incisor/lateral incisors [5]. This should be taken into consideration when selecting the site of mini-implant placement.

Stability of the mini-implant is also influenced by several other factors. Miyawaki [1] found that low mandibular plane angle has a high success rate followed by average and high mandibular plane angles. Skeletal class III malocclusion has a high success rate followed by class II and class I patterns. The success rate was higher in females than in males and in the absence of crowding, periodontitits and TMD symptoms. It was also higher in the absence of inflammation, in self-drilling mini-implants compared to self-tapping mini-implants and in the maxillary arch compared to the mandibular arch. All the above factors were confirmed by Kuroda [4] and Park [2]. However, Miyawaki’s study [1] showed that the success rate was highest in patients between 20 and 30 years of age followed by those above 30 years and less than 20 years, while Kuroda et al. [4] found the success rate to be highest in individuals more than 30 years followed by those less than 20 years with the least being 20 to 30 years.

Park [2] found that implant with 1.2-mm diameter had a higher success rate compared to a 2-mm mini-implant. The left side had a higher success rate compared to the right side. He suggested that placement of the mini-implant high in the upper oral mucosa had a greater success rate compared to a lower level in the upper oral mucosa and upper attached gingiva. The 30° to 40° angulation had a higher success rate compared to the 90° and 10° to 20°.

An angulation of 30° to 40° [11-14] has also been proposed by several other authors to increase the surface contact between the mini-implant and the cortical bone. An angulation of 20° has been suggested by Wilmes [8]. A greater angulation can result in increased stress during placement [8] and removal of implant [15] because of the greater amount of cortical bone the mini-implant has to penetrate [8].

Some of the precautions to be taken in areas with minimal interradicular bone would be to angulate the mini-implant to the long axis of the tooth towards the root apex to reduce proximal contact with the adjacent teeth. A 1.2-mm-diameter mini-implant can be used compared to a mini-implant with 2-mm diameter. The conical-shaped mini-implant has tight contact to the adjacent bone tissue and has good primary stability compared to cylindrical mini-implants [16]. Also, the contact between the adjacent teeth can be used as a guide for mesiodistal positioning of the mini-implant. Interproximal root contact can be minimised by placing the mini-implant parallel to the contact of the adjacent teeth [10] or by placing the mini-implant with a 10° mesial angulation to the buccal surface of the teeth.

All the above factors play a major role in the clinical success of the mini-implant. A stent designed taking the above factors into consideration will enhance the primary and secondary stability of the mini-implant. The stent described above is simple and easy to fabricate. It allows three-dimensional control in all three planes of space. Removal of the stent is also easy. Depending on the site of mini-implant placement, the length of the horizontal limb can be adjusted to place the ‘u’ exactly between the roots of the adjacent teeth or 1 mm anterior or posterior to the midline if protraction or retraction of the dentition is desired. The vertical limb can also be adjusted to place the mini-implant in a high, medium and low position. The stent can be modified for placement in different areas of the maxilla and mandible.

A number of stents have been proposed by the several clinicians. The stent described by Jae-Jung Yua et al. [17] requires the use of cone beam computed tomography and hence very expensive. It also carries risk of radiation exposure. The stent described by Seong-Hun Kim et al. [18] is a stereolithographic surgical guide and hence expensive and difficult to fabricate for routine use. The three-dimensional stent described by Eduardo et al. [19] is prefabricated in three vertical heights 5, 7 and 9 mm as provided by the manufacturer and cannot be made chairside for a specific height. Cousley et al. [20] described six standard custom guide made for Infinitas mini-implant. The stent described by Kravitz et al. [21] consists of an anterior simplified stent which does not take into consideration the angulation of mini-implant placement. Also, as the wire is placed flush with the bracket slot, it gives a wide area for mini-implant placement and hence a greater chance of root contact. Also, the placement is restricted only to the anterior region. The stent described by Barros et al. [22] requires elaborate inventory and that proposed by Camillo Morea et al. [23] involves elaborate laboratory work.

An attempt has been made in this paper to fulfil all the requirements of an ideal stent and also overcome the drawbacks of the existing methods during mini-implant placement.

### Conclusion

This stent is simple, easy to fabricate, cost effective, provides ease of insertion and removal and also provides three-dimensional orientation of the mini-implant.

## Competing interest

The author declares no competing interest.
